# Solution Oligonucleotide APIs: Regulatory Considerations

**DOI:** 10.1007/s43441-022-00384-2

**Published:** 2022-02-08

**Authors:** Christian Wetter, Chris Chorley, Corrine Curtis, Nicole del Canto, J. Gair Ford, Jennifer Franklin, Cinzia Gazziola, Michael T. Jones, Judy Lee, Arnold McAuley, Florence C. E. Saraber, Audrey Scott, Janine Tom

**Affiliations:** 1 Pharma Technical Regulatory, F. Hoffmann – La Roche AG, Grenzacherstrasse 124, 4070 Basel, Switzerland; 2 Global Regulatory CMC, Biogen Idec Limited, Innovation House, 70 Norden Road, Maidenhead, SL6 4AY Berkshire UK; 3 Global Regulatory Affairs CMC, Janssen Research and Development, 965 Chesterbrook Blvd, Wayne, PA 19087 USA; 4 Global Regulatory CMC, Biogen, Inc., 225 Binney Street, Cambridge, MA 02142 USA; 5 CMC Regulatory Affairs, AstraZeneca, Charter Way, Macclesfield, SK10 2NA UK; 6 CMC Regulatory Affairs, Ionis Pharmaceuticals, Inc., 2855 Gazelle Court, Carlsbad, CA 92010 USA; 7 Pharmecuetical Sciences, Pfizer Inc., 875 Chesterfield Parkway West, Chesterfield, MO 63017 USA; 8 Regulatory Affairs CMC, Novartis Pharmaceuticals Corporation, One Health Plaza, East Hanover, NJ 07936-1080 USA; 9 Process Development, Amgen, Inc, Thousand Oaks, CA 91320 USA; 10 CMC Leadership and Dossier Development and Operations, Janssen Biologics B.V., Einsteinweg 101, 2333 CB Leiden, The Netherlands; 11 Development Projects - CMC Regulatory Affairs, GlaxoSmithKline Research and Development, Park Road, Ware, SG12 0DP Hertfordshire UK

**Keywords:** Oligonucleotides, Solution API, Formulated API, API mix, Regulatory

## Abstract

Manufacture of oligonucleotide active pharmaceutical ingredients (APIs) typically consists of solid-phase synthesis, deprotection and cleavage, purification and filtration, and isolation from aqueous solutions through lyophilization. In the first step of drug product manufacture, the API is dissolved in water again and excipients are added. While isolation of oligonucleotide APIs can be meaningful in many cases, there may be cases where keeping the API in solution provides benefit, and multiple technical aspects must be taken into account and balanced when determining the appropriate API form. A significant factor is whether an API in solution will contain additional components. While APIs in solution containing additional components (so-called formulated APIs) are well established for biological products, there are regulatory guidelines in place that represent hurdles for industry to using a formulated API approach for oligonucleotide drugs. The present communication outlines conditions where a formulated API approach can be chosen in compliance with existing guidelines. Relevant aspects pertaining to risk management, GMP standards, facility design, control strategies, and regulatory submission content are discussed. In addition, the authors propose that existing guidelines be modernized to enable the use of a formulated API approach for additional reasons than the ones described in the existing regulatory framework. The manuscript aims to promote a dialog with regulators in this field.

## Introduction

Muslehiddinoglu et al. described the concept of active pharmaceutical ingredients (APIs) in solution for oligonucleotides [1]. The publication discusses technical aspects related to solution oligonucleotide APIs, such as their stability, manufacture, microbial control, packaging, and storage. Various criteria, such as supply chain, scale, and dosage strengths, may influence the best choice of the API form—resulting in use of either a solid API or a solution API. The solution API may be ready to fill or may require further dilution or addition of excipients. Muslehiddinoglu et al. left regulatory aspects out of scope of the published article; therefore, the present communication aims to complement the publication and discuss relevant regulatory and GMP aspects of solution APIs.

Within the class of solution APIs, the authors distinguish between APIs in water (potentially containing required processing salts) and formulated APIs. APIs in water (with required processing salts) should be considered as a conventional API form. Their designation as an API is considered compliant with existing guidelines. Formulated APIs that show stability benefits can also be considered as APIs in compliance with existing guidelines.

At this moment, if chosen for workability reasons alone, the European Medicines Agency (EMA) and Health Canada consider formulated APIs as drug product intermediates. The authors would like to use EMA’s initiative ‘Regulatory Science to 2025—Strategic Reflection’, specifically in “developing expertise in novel manufacturing technologies” to challenge this assumption. The authors propose that existing guidelines are modernized and allow the designation of formulated oligonucleotide APIs as APIs for additional reasons, such as benefits related to (greener) manufacturing, supply security, quality, and other science-based reasons [2].

It will be the responsibility of the applicant to evaluate the various aspects discussed by Muslehiddinoglu et al. [1] and to use the aspects discussed in this paper for justification of the selected approach and the proposed API designation.

The current article focuses on oligonucleotides, but the considerations provided could also be applied to other small-molecule modalities administered via a parenteral route, such as peptides.

## Discussion

### Problem Statement

Oligonucleotide APIs are typically synthetically derived and can be isolated as a lyophilized solid, in solution in water, or as formulated API. While available guidelines generally provide flexibility with respect to the designation of a formulated API as an API, the EMA and Health Canada have set a more restrictive framework in which API mixes can be designated as APIs in exceptional cases only and the standard is to designate API-mixes as drug product intermediates. A review of applicable guidelines is provided below.

When a formulated API is required to be designated as a drug product intermediate, manufacturers must fulfill drug product GMP standards for the manufacturing steps converting the API into a formulated API. The requirements for clean areas as defined in ISO 14644-1 [3] and the EU guideline to GMP, Annex 1 [4] would thus apply. Compliance to these standards is typically not foreseen in facilities designed for manufacture of oligonucleotide APIs. One reason for this is that there are often competing requirements between clean areas and worker safety in chemical manufacture and this makes implementation of sterile drug product GMP standards complex.

A further challenge of designating a formulated API as a drug product intermediate is related to the assignment of the shelf-life. According to standard requirements for chemical products, the shelf-life is defined by the start of the drug product manufacturing process, i.e., the mixing of the API with other product ingredients [5]. Storage of the formulated API thus becomes part of the drug product shelf-life, and storage for a prolonged period before further manufacture and/or filling would therefore shorten the remaining shelf-life of the drug product upon release for market distribution. This would require more frequent manufacture and smaller formulated API batches.

In addition, if formulated API storage is part of the product shelf-life and no retest period can be assigned, this can be a problem for low volume products where flexibility in the supply chain is needed. Manufacture of small drug product batches on a need basis requires storage of the API over a long period and retesting the API multiple times. If the formulated API is a drug product intermediate, retesting is not possible. This would result in more disposed product or more frequent and smaller API manufacturing campaigns.

Similar challenges have been identified when using co-processed APIs [6].

### Current Regulatory Landscape

The guideline review provided below indicates that existing ICH and WHO guidelines support the designation of formulated APIs as APIs. A more restrictive framework is provided by the EMA and Health Canada, which leads to divergence in regulatory expectations. A summary of the relevant guidelines is provided in Table 1 below.

**Table 1:** Summary of guideline review

Guideline issuing body	Guideline	Relevant guideline text
ICH	Q7, Glossary [7]	An API is defined as “any substance or mixture of substances intended to be used in the manufacture of a drug (medicinal) product (…)”
	Q7, Q&A [8]	“When a mixture is classified in the regulatory filing as an API in a region or country in which it is used in a drug product, ICH Q7 should be applied to the manufacturing of these mixtures [ICH Q7, Sect. 1.2, 20—see Glossary for definition of ‘API’]”
	Q6B [9]	For biological products, the drug substance (bulk material) “may also contain excipients, including other components, such as buffers”
WHO	Points to consider for development of finished products [10]	An API is defined as “Any substance or mixture of substances intended to be used in the manufacture of a pharmaceutical dosage form and that, when so used, becomes an active ingredient of that pharmaceutical dosage form”
EMA	Q&A document on API mix [11]	The CHMP quality working party (QWP) defines an API mix as a “mixture of an API with one or more excipients”, and “the manufacture of an API mix is considered to be the first step of the manufacture of a finished product”. Further, “in certain circumstances, i.e., stability or safety reasons, the applicant can submit data on such a mixture under part 3.2.S (…).” However, “In case of an API mix prepared due to workability purposes or reasons other than safety and stability, the manufacturing steps from the addition of the excipient to the API should be described in the appropriate part of CTD 3.2.P. In addition, the steps following addition of the excipient must be conducted in accordance with GMP Part I and an appropriate manufacturing authorisation”. Moreover, “a justification based only on workability reasons, e.g., to ease handling when processed into final dosage form, is not acceptable. Toxicological considerations (e.g., very potent drugs) fall under workability reasons and are not accepted as justifications.”
Health Canada	Quality guidance for (abbreviated) new drug submissions [12]	Health Canada states the following related to the start of drug product manufacture and possible exceptions based on safety and stability reasons: “That first processing step of the drug substance in the presence of any other substance would be considered a drug product manufacturing activity, subject to Part C, Division 2 of the Food and Drug Regulations, and would define the date from which the expiry date for the drug product would be established. (…) Sponsors having situations that might be an alternative to the above interpretation (e.g., inability to isolate the drug substance in a pure and stable form or mixing with excipients for safety or stability purposes, e.g., nitroglycerin, cholecalciferol) should discuss their case and scientific justification in advance with the pre-market approval bureau/office.” The expectations are aligned with the QWP Q&A discussed above, and Health Canada encourages a discussion with their pre-market approval bureau/office

### Unique Composition Features of Oligonucleotide APIs

Oligonucleotide APIs in water should not be considered as API mixtures, but should be designated as APIs. The oligonucleotide in water can be isolated as such from the API manufacturing process, and hence water is not added as a first (and new) component to the final API. An oligonucleotide API in its solid state can contain up to 20% of water and water is an integral part of the nucleic acid structure [13]. Therefore, an oligonucleotide API can be considered as an amorphous hydrate [14], supporting that water does not have to be considered as a first component added.

Not only should APIs in water be designated as APIs, but also APIs in water containing processing salts should be designated as APIs as well. In fact, downstream processing following oligonucleotide synthesis may afford an API in solution containing certain required processing salts, e.g., phosphates. Examples could include use of a buffer to control pH or utilization of salts from a previous chromatographic step to maintain adequate flux during ultrafiltration/diafiltration (UF/DF). These components are often also part of the drug product formulation and therefore it should not be necessary to remove them from the API in solution, and they would consequently be part of the final composition. These components are not used in their function as excipients alone. Therefore, an API with processing salts in water, requiring further compounding and/or dilution at the drug product stage should be designated as an API.

### Proposed Path Forward

The authors recognize that two elements need to be addressed to enable the designation of formulated APIs as APIs according to those in existing guidelines: First, designation of an API mix as an API should not be justified solely by the benefits from a workability perspective [11, 12]. Second, applicants should address concerns related to the GMP standards of API manufacturing facilities [3, 4].

### Stability Considerations

The designation of formulated APIs as APIs is a generally accepted practice for biological products, justified on the basis of the instability of proteins. Oligonucleotides are generally stable in formulation; however, certain factors can impact stability. For example, the pH of the formulation strongly influences oligonucleotide degradation [15–17] and the addition of buffers to an oligonucleotide API can have a stability benefit. For example, Poecheim et al. showed in 2018 that buffered solutions of a model oligonucleotide API show superior stability behavior compared to the aqueous solution [18]. The stability promoting properties conferred by a buffer support the use of formulated oligonucleotide APIs as a meaningful option.

In order to address the requirements defined in the QWP Q&A, stability data should be generated for both the API (in solid state or in water) and the formulated API under ICH long-term conditions for up to 6 months. If it can be demonstrated that the formulated API provides improved stability behavior, the designation of the formulated API as an API should be permissible. In case the stability behavior is equivalent, the authors propose that additional aspects can be considered for justification, as detailed below.

If the API is isolated as a solid, the solid API can be used for the stability comparison with the formulated API. If the API is processed further as an aqueous solution, without isolation, the API in water can be used for comparison with the formulated API. Another reason of selecting the API in water as a comparator could be the hygroscopic nature of oligonucleotide APIs discussed above, i.e., in order to be able to make an accurate comparison of the relative assays, it may be better to avoid weighing out a solid API and to use API in water instead.

### Control Strategy, GMP, and Facility Considerations

In addition to the stability and composition considerations detailed above, a comprehensive control strategy has to be implemented for the manufacture of the formulated API. The control strategy will mitigate potential risk inherent to the management and addition of components (i.e., buffer systems, tonicity agents) as part of the formulated API manufacturing process. The elements of the control strategy should include design of the manufacturing process, e.g., closed or controlled manufacturing systems, design of manufacturing facilities and utilities, and implementation of appropriate quality systems (“fit-for-purpose approach in application of GMP” [2]). The control strategy—particularly in an enhanced development approach—should be complemented with risk assessments and implementation of risk mitigation activities (“use of risk-based approaches to manufacturing processes and control strategies” [2]), and an evaluation of process validation results to ensure sufficient controls are in place. In totality, the control strategy should demonstrate delivery of safe and efficacious product. In detail

- API manufacturing process risk assessments should cover the whole process down to the formulated API: High-risk parameters need to be effectively mitigated. The downstream process must be adequately characterized and controlled to consistently produce API meeting its quality targets.
- Microbial controls incorporated into the process should assure consistent delivery of low bioburden, low endotoxin-formulated API appropriate for further aseptic processing yielding a sterile drug product. The microbial control strategy should include elements of facility design, process controls, personnel requirements, environmental and utility monitoring systems, and procedural responses to contamination.
- Components used in the manufacture of the formulated API should be controlled as critical raw materials. Following the standards defined in EU Directive 2001/83/EC, Annex I, Sect. 3.2.2.4 [19], “the specifications and their justifications shall be detailed. The analytical procedures shall be described and duly validated.” The components should be handled in a facility offering an appropriate clean area concept as per ISO [3] and EU guidance [4].
- In compliance with the EMA guideline on water for pharmaceutical use, dated 2020, [20], it is proposed to use water for injection (WFI) starting with the step where the water stays in the drug product, e.g., UF/DF. The steps upstream need not use WFI.

The requirements for formulated APIs detailed above would provide sufficient controls to permit their production at the site of API manufacture.

### Additional Benefits Serving as Justification

In the case of equivalent stability behavior (see above), it should be permissible to use additional benefits of a formulated API approach as justification for designation as an API. Specifically, advantages such as reduced environmental impact (“support the development of greener manufacturing technologies” [2]), improved quality and supply security, more innovative manufacturing, and other benefits as described in the paper by Muslehiddinoglu et al. in 2020 [1] could also be considered when justifying the chosen formulated API approach.

Consequently, the authors propose that existing guidelines are modernized, providing more flexibility and allow the designation of formulated or solution oligonucleotide APIs as APIs for additional reasons than those two currently permissible.

### Regulatory Documentation

To promote alignment in the regulatory documentation, some selected aspects are discussed and the location of the corresponding information in module 3 of the CTD is provided in Table 2. Other locations in the application may be possible in alignment with ICH M4Q [21]. Some definitions of terms used in the paper are provided in Table 3.

**Table 2:** Formulated API-specific information and location in module 3 of CTD

CTD section	Content	Comment
Module 1, Labeling and Prescribing Information (PI)	Processing salts used to manufacture the API and remaining in the product, components used in the manufacture of the formulated API, as well as potential additional excipients introduced in the drug product manufacture before filling are required to be listed on the label and in the prescribing information (PI)	In countries where the country of origin has to be provided on the label and PI, only the country of origin of the (formulated) API will be provided; the country of origin of the components/excipients will not be provided, as the API is defined as the molecule of action
3.2.S.2.2	Process description, from the starting materials down to the isolation of the formulated API	Processing salts that remain in the drug product and components that are part of the formulated API should be listed in 3.2.P.1 [11]. Their contribution to the drug product composition should be provided
3.2.S.2.3	Specifications of the materials used to manufacture the formulated API should be provided in this section. This includes processing salts used in the API manufacture that remain in the product and the components added to the API, yielding the formulated API If components are designated as critical raw materials, then the specifications and their justifications should be detailed. In addition, the analytical procedures should be described and duly validated	Processing salts and components and reference to relevant standards (i.e., in-house monographs) will also be provided in 3.2.P.1
3.2.S.2.6	Justification of the formulated API approach, including comparative stability data demonstrating improved behavior of formulated API compared to API in solid state/in water should be provided in this section. Other justifications could also be included in this section A summary of the API manufacturing process, process characterization studies to ensure process robustness, including API formulation consistency and microbial attributes should also be included, as well as extractable and leachable substances (if in bags), endotoxin clearance, component selection, and API container closure integrity. Particularly in enhanced development, risk assessment and risk management will be an important component of the application	Tight linkage with the discussion provided in 3.2.P.2
3.2.S.3.1	Information on characterization of the API, generated at the stage where it is most suitable, must be provided. For certain parameters, characterization using the solid API may be preferred, whereas for other parameters using the formulated API may be appropriate	A discussion should be provided if processing salts and components added to the formulated API have an impact on the characterization
3.2.S.4.1	Release testing should be performed using the formulated API. The formulated API should be analyzed for identity and content (or relative concentration) of components used in its manufacture	As proposed above in the section on characterization, certain release tests may be performed upstream in lieu of testing of the formulated API Release tests that cannot be performed on the formulated API should be “included on the specification and confirmed through upstream controls (e.g., as in Real-Time Release Testing (RTRT))” [22] The control strategy should be justified appropriately, e.g., in 3.2.S.4.5
3.2.S.7.x	Re-test period and storage conditions will be assigned to the formulated API, justified by stability data of the formulated API	Stability testing will be performed on the formulated API Comparative stability data are provided in 3.2.S.2.6
3.2.P.1	Composition of the drug product should be provided in this section, including excipients used in the drug product manufacture, as well as processing salts and components added when manufacturing the formulated API (which remain in the product)	Processing salts and components used in the manufacture of the formulated API contribute to the composition of the finished product. They should be quantified in 3.2.P.1 and should be listed on the label and in the PI. For processing/buffer salts, it may be appropriate to state that a sufficient quantity to achieve a certain pH is used
3.2.P.2.1	Justification of the choice and levels of excipients used in the drug product manufacture should be provided in this section	Discussion expected to be linked to discussion in 3.2.S.2.6 (e.g., for selected processing salts and components)
3.2.P.2.5	Microbial attributes and control for drug product manufacture should be discussed in this section	This section should be written with a close consideration of the information provided in 3.2.S.2.6
3.2.P.3.2	The batch formula of drug product manufacture, starting from the formulated API, should be provided	Formulated API amount, any drug product excipients, and/or WFI added as part of drug product manufacture
3.2.P.3.3	A description of the drug product manufacturing process should be provided	The drug product manufacturing process consists of the potential addition of excipients to the formulated API, sterile filtration, and aseptic filling. Thawing/equilibration may not be needed if formulated API can be stored at room temperature The start of the drug product manufacture should be defined by the introduction of the formulated API into further processing steps, i.e., with the potential addition of excipients required for the formulation and/or prior to the sterile filtration and aseptic filling. The start of drug product manufacture is also the proposed start of the drug product shelf-life
3.2.P.8.1	The definition of the drug product shelf-life and the storage condition should be justified	Stability testing will be performed on the drug product Shelf-life starts with the first step of the drug product manufacturing process, i.e., the use of the formulated API

**Table 3:** Definitions

Term	Definition
Active pharmaceutical ingredient (API)	In the context of this paper, an API can be a solid API or the API in water with or without processing salts. The processing salts are used in and are needed for the API manufacturing and purification process, e.g., for the UF/DF step Depending on the jurisdictions the terms active substance or drug substance are preferred. In the context of this paper, the three terms can be used interchangeably
Drug product intermediate	A drug product intermediate is described in 3.2.P.3.4. Formulated APIs are not considered as drug product intermediates When a formulated APIs is used, manufacture and holding of drug product intermediates are unlikely
Excipients	Excipients are added to the formulated API during drug product manufacture, before final filtration and fill/finish. Excipients added during formulated API manufacture are called components
Component of the formulated API	A component is a buffer salt or a tonicity agent that is part of the formulated API. A component is added to the API in solution after the final purification or filtration step. The component will typically appear in 3.2.P.1 and on the label/PI together with the processing salts and excipients
Formulated API	A formulated API is a mixture of the API in water with processing salts and components and will be presented in 3.2.S of the marketing application
Processing salts	A processing salt is used in and needed for the API manufacturing or purification process and will not be removed in the last purification or filtration step yielding the API. The processing salts will typically be listed in 3.2.P.1 and on the label/PI together with the components of the formulated API and excipients

## Summary and conclusion

The use of formulated APIs is generally accepted for biologics products. For oligonucleotides the existing regulatory framework is more restrictive and formulated API approaches can be justified in compliance with existing guidelines only when improved stability can be demonstrated. The authors propose that additional justifications should be permissible, e.g., to enable greener and more innovative manufacturing. This paper provides recommendations for development, relevant standards, as well as justification and information to be provided in regulatory documentation to support the use of formulated oligonucleotide APIs.

### Outlook

The EPOC group welcomes interaction and discussion with regulators to promote the use of the formulated API approach in oligonucleotide drug manufacture. Further, the authors recommend that relevant guidance documents be modernized so that they better reflect the specific needs of therapeutic oligonucleotide manufacture, as outlined in this paper.

## Conflict of interest

The authors are employees and stock shareholders of the individual EPOC member companies. The authors are of the opinion that the reported relationships do not affect their ability to write an unbiased manuscript.
